# The diaspora model for human migration

**DOI:** 10.1093/pnasnexus/pgae178

**Published:** 2024-05-21

**Authors:** Rafael Prieto-Curiel, Ola Ali, Elma Dervić, Fariba Karimi, Elisa Omodei, Rainer Stütz, Georg Heiler, Yurij Holovatch

**Affiliations:** Complexity Science Hub, Vienna, Austria; Complexity Science Hub, Vienna, Austria; Complexity Science Hub, Vienna, Austria; Complexity Science Hub, Vienna, Austria; Vienna University of Technology (TU Wien), Vienna, Austria; Graz University of Technology (TU Graz), Graz, Austria; Department of Network and Data Science, Central European University, Vienna, Austria; Complexity Science Hub, Vienna, Austria; Complexity Science Hub, Vienna, Austria; Complexity Science Hub, Vienna, Austria; Institute for Condensed Matter Physics, National Academy of Sciences of Ukraine, Lviv, Ukraine; L4 Collaboration & Doctoral College for the Statistical Physics of Complex Systems, Lviv, Ukraine; Centre for Fluid and Complex Systems, Coventry University, Coventry, UK

**Keywords:** migration, diaspora, gravity, Austria, United States of America

## Abstract

Migration’s impact spans various social dimensions, including demography, sustainability, politics, economy, and gender disparities. Yet, the decision-making process behind migrants choosing their destination remains elusive. Existing models primarily rely on population size and travel distance to explain the spatial patterns of migration flows, overlooking significant population heterogeneities. Paradoxically, migrants often travel long distances and to smaller destinations if their diaspora is present in those locations. To address this gap, we propose the diaspora model of migration, incorporating intensity (the number of people moving to a country), and assortativity (the destination within the country). Our model considers only the existing diaspora sizes in the destination country, influencing the probability of migrants selecting a specific residence. Despite its simplicity, our model accurately reproduces the observed stable flow and distribution of migration in Austria (postal code level) and US metropolitan areas, yielding precise estimates of migrant inflow at various geographic scales. Given the increase in international migrations, this study enlightens our understanding of migration flow heterogeneities, helping design more inclusive, integrated cities.

**Significance Statement**

Understanding migration is critical for infrastructure planning and policy in an era of increasing global movement. Most migration models focus only on where migrants decide to go but ignore the number of people that move. Here, we introduce a new model that dissects migration into intensity (number of migrants) and assortativity (their destination choices). We show that a country’s existing immigrant community size can predict migration intensity, and migrants often choose destinations with these established communities. It also highlights a challenge: migrants cluster in specific areas, creating unintentional cultural walls. Our work equips policymakers with essential insights for enhancing infrastructure readiness and promoting urban cohesion.

## Introduction

Births, deaths, and migration are the most relevant demographic components of population change, but migration is the most difficult to quantify, model, and forecast (1). Mass movements of people change the spatial distribution of the population and explain why some places grow faster than others (2, 3). The size of cities and hierarchy are heavily affected by migration (4–8). Today, international migrants would form the fourth largest country in the world, and approximately 1% of the World’s GDP is sent as international remittances (9, 10). Migration is a complicated process driven by economic and demographic factors (11). Migration is a selective process that tends to attract young and highly skilled people into large cities, increasing the burden of human capital flight but easing economic disparities across borders (12–14). It is a core strategy for coping with unemployment, violence, or disasters (15–19). Migration eases the pressure of an ageing population and alters the gender imbalance (20). Accurately predicting the number of individuals who will relocate and their precise destinations holds significant implications for resource allocation, planning, and hosting strategies. Yet, most migration models have many critical weaknesses. Migration models often focus on where they will go but ignore a more critical question: how many will move. Also, they try to capture the attractiveness of a destination based on population size, so they tend to fail drastically at small spatial units, such as neighborhoods. Finally, they distinguish migrants only by location on a map, so they fail when comparing many origins. Here, we construct a migration model with the aim of forecasting that distinguishes between the nationality of migrants and works accurately at the neighborhood level.

Critical repeated patterns emerge when studying thousands of migrations. For example, most migration is across short distances, between nearby areas (21–24). Also, large destinations attract disproportionally more migrants than smaller ones (25, 26). The gravity model for human migration has been extensively used to capture both the impact of size and of distance simultaneously (27–29). However, the gravity model has many limitations, including parameter dependence, issues with estimation, and bias, among others (30–32). Extensions of the gravity model have been constructed by looking at job opportunities, the distribution of points of interest, the road network, and other geographical attributes (33–39). There are two main drawbacks to these models. First, they ignore the vast heterogeneity of migrants and distinguish individuals only by their location. As long as two individuals are at a similar distance to some destination, the gravity model (and its extensions) will give identical migration estimates. Thus, for these types of models, what makes a person different from others is only their location. Second, the gravity model and its extensions focus only on where migrants decide to go (using elements such as city sizes or job opportunities) but usually take the actual number of people that move as a given quantity. Yet, we observe that migration is a much more complex process that depends on many more aspects. The reasons why a person leaves their home in Nicaragua are not similar to a person leaving their home in Italy or Senegal, and the same applies to destinations. Silicon Valley attracts a different kind of people than Las Vegas or Hollywood. A city’s push and pull factors do not apply equally to all people (40, 41). At a microscopic level, a similar process observed with residential selection profoundly impacts society, where mild preferences for living with similar neighbors may cause severe segregation and create politically homogeneous neighborhoods (42–45). Likewise, if migrants prefer to move to some community, they will naturally form a significant diaspora in the new destination. However, models do not capture the spatial sorting of individuals.

Various reasons explain why people with different backgrounds are attracted to distinct places. While numerous reasons could explain why an individual migrates to a new destination, we observe surprising regularities and predictability in migration flows. One explanation is related to the flow of information. Individuals are more attracted to places where they have more information, mainly acquired from their social networks. In this case, early migrants reduce uncertainty and provide adequate information for late arrivals, creating a self-reinforcing mechanism (22, 46–49). The observed pattern could also be due to repeated factors that attract people with similar conditions. For example, an expensive neighborhood receives wealthier people, students tend to move to university towns, startups attract engineers, or cities such as Los Angeles attract artists. Another explanation could be related to migration’s emotional and monetary costs. Moving to a place with more migrants can lower the barriers to integrating into a new society and the legal and administrative costs involved (50, 51). Most people move to places with pre-existing ties (52–55). This process relates to one of the most fundamental forces of our social life, namely homophily, the tendency to interact with similar others (56). Group identity could be based on race and ethnicity, leading to homophily and affinity between people (57). Therefore, homophily can directly or indirectly affect people’s decision to migrate to a specific neighborhood. A prime example of such affinity is migration and co-location due to strong same-race and same-ethnic dating or marriage preferences (58, 59). However, it is worth considering that other characteristics, such as shared language or a common place of origin, might also play significant roles in fostering strong social ties among people in a foreign environment, leading to the creation of diasporas.

This study examines the influence of homophilic preferences on international migration. We use the term *diaspora* to refer to a group of people from one nationality living elsewhere. Consequently, we distinguish individuals not solely by their geographic location but, more significantly, by their country of origin. Thus, diaspora encompasses similarities in race, ethnicity, and more. We will see that the diaspora is a much more accurate explanation for modeling and predicting future migration beyond factors like distance or a city’s population. Many social processes tend to be highly homophilic, and migration is no exception.

Here, we construct the diaspora migration model. The contribution is a novel model based on the pull impact of the diaspora. Instead of looking at population size, travel distance, or points of interest, our model uses only the diaspora size. We analyze two migration scenarios. First, we look at population registers in Austria and explore arrivals to the country from other parts of the world (details in the Supplementary Material A). Second, we use the international arrivals to metropolitan areas in the United States of America to show the pull mechanism of diasporas (Supplementary Material D). We show that migration is a highly homophilic process. Opposing the principles of the gravity model, migrants travel long distances and go to small cities if there is a sizeable diaspora in the destination. We estimate that diasporas have a pulling impact, where 10,000 individuals will attract roughly 1,204 new arrivals yearly in the case of Austria. We show that diaspora size can accurately explain migration even at the neighborhood level. The diaspora model is more precise than the gravity model, holding for both international arrivals at the postal code level in Austria and the metropolitan area level in the United States of America.

## Results

### Diaspora migration model

**Fig. 1.**
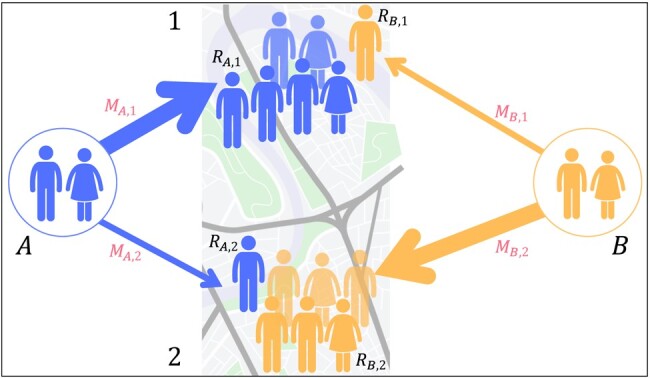
Illustration of the diaspora migration model. We divide migration into two separate components: intensity (related to the arrival of migrants) and assortativity (related to where migrants decide to go). The diaspora model uses the size of the pre-existing population (depicted as the people on the map with different colors to represent people with different backgrounds) to estimate a steady inflow of migrants (represented by the arrow thickness) and their distribution across two regions (marked as 1 and 2 in the map).

Although there are many reasons why a migrant from some country chooses to move to some destination, similar reasons applied in the past to previous migrants from that country. Instead of observing and modeling the reasons, the principle we apply here is to look at how many people were already attracted to some destination and use it as a proxy to forecast future migrants (Fig. 1). For a given destination, we model migration with a hierarchical model, where the first component captures how many people will arrive, and the second is deciding where they will move to (60). The first component is the *intensity* of the migration flow (6, 61, 62). The intensity of arrivals from country *i* is modeled with a homogeneous rate $\lambda_{i}$. Thus, the hosting country expects $\lambda_{i}$ migrants from *i* daily. The second component is the *assortativity*. Once people decide to move, they choose *j* destination among *ν* options in the hosting country (for example, among *ν* metropolitan areas or neighborhoods) with a multinomial distribution. The probability that a person arriving from *i* goes to *j* is $\pi_{ij}$, with $\sum_{j}\pi_{ij}=1$. Combining the two components—intensity and assortativity—the arrivals to destination *j* have a homogeneous rate $\lambda_{i}\pi_{ij}$. In essence, we model first how many people will move to a country and then how they choose a specific destination (Supplementary Material B). We show that arrivals to a country can effectively be modeled by a constant rate, which depends on the country of origin (see the Methods section).

We capture the degree to which migration is a homophilic process by looking at the size of the diaspora (Fig. 1). We assume that $\lambda_{i}$ (which is a country-specific rate) can be expressed as $\rho R_{i}$, where $R_{i}$ is the total diaspora from country *i*, so the arrival rate to the country depends only on the size of the diaspora and a *pull rate ρ* that applies equally to all countries. Then, we assume that the assortativity can be expressed as $\pi_{ij}\propto R_{ij}$, where $R_{ij}$ is the diaspora from country *i* in destination *j* (so $R_{i}=\sum_{j}R_{ij}$). Combining both assumptions, arrivals from country *i* to location *j* have a rate $\rho R_{ij}$. Consequently, the expected number of migrants from *i* to destination *j* for *t* days, is


(1) $$M_{ij}(t)=\rho R_{ij}t,$$

reflecting that arrivals depend on the size of the diaspora (details in the Methods section). Ignoring other demographic processes (births and deaths), the diaspora model results in the conservation of assortativity, meaning that the way migrants have been distributed in the past among neighborhoods will be the observed pattern in the future. Thus, within the time window considered, assortativity is stable (Supplementary Material A).

### Austrian migration dynamics

Migration data are often scarce and requires long observation periods to distinguish between migration and other types of mobility (63, 64). Here, we use individualized data, which captures the primary residence of all foreign-born individuals in the country. Population registers capture all address changes and have become the primary source of migration data (6, 65, 66). Population registers corresponding to all arrivals to Austria before a fixed date (December 2022) and 200 days later (labeled as “arrivals”) are used to test the model. The data contain information regarding 1.46 million foreign-born individuals living in Austria, and it is used to determine the size of the diaspora of all countries at the postal code level, $R_{ij}$ (Supplementary Material A).

**Fig. 2.**
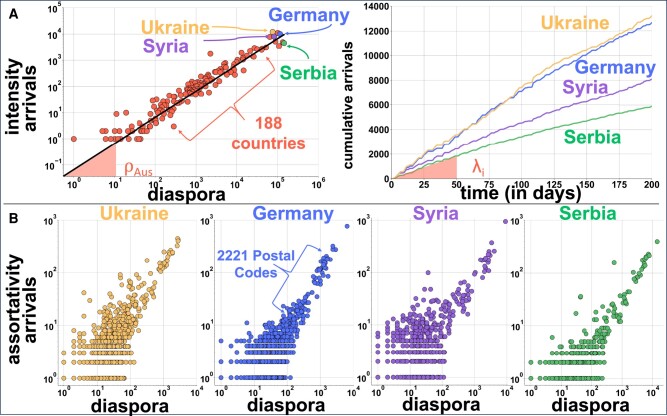
Observed intensity and assortativity of migrants. A)—(Left) The intensity of arrivals versus the pre-existing diaspora size. Each disc represents a country of origin, the diaspora size is the number of existing migrants from that country of origin in Austria before December 2022, and the arrivals is the number of new migrants observed after 200 days. The black line is the daily pull rate of $\rho_{\mathrm{Aus}}$. We highlight arrivals from Ukraine, Germany, Syria, and Serbia because they belong to countries of origin with the biggest diaspora size in Austria and have different economic and political backgrounds and migration histories (Supplementary Material A). (Right) Observed cumulative arrival of the top diasporas within our observation period, with different arrival rates $\lambda_{i}$. B)—Assortativity of migrants from the top diasporas. Each point represents a postal code in Austria (other diasporas in Supplementary Material A).

Arrivals to Austria are used to quantify the intensity and assortativity of the migration flow. In total, 111,244 individuals arrived in the country during the 200 observed days. The daily pull rate is around $\rho_{Aus}=3.29\times 10^{-4}$ per person (see the Methods section). Hence, we expect one arrival for every $1/\rho_{\mathrm{Aus}}\approx 3,031$ people, and the same applies to any diaspora and any destination considered (Fig. 2). The diaspora model estimates the number of arrivals from any country to any destination at granular levels (data for each destination is shown by a disc in Fig. 2b). It gives consistent estimates for geographic units (neighborhoods) that can be aggregated to more extensive areas (such as cities or provinces).

**Fig. 3.**
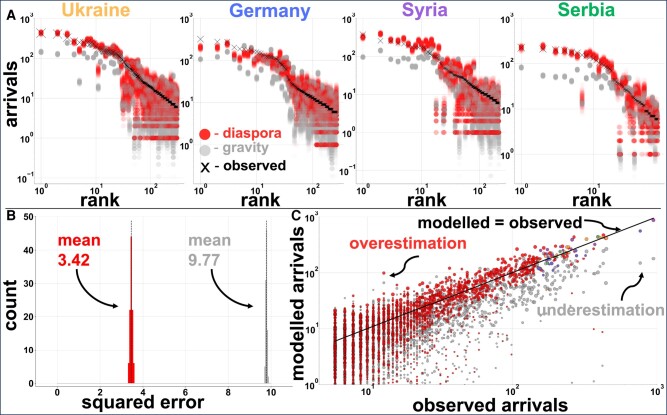
Model results and error estimation. A)—Results of the diaspora model for migration (red) compared to the gravity model (grey) over 100 runs and the empirical observations (black crosses). The opacity of the discs is intentionally low to account for overlapping points, indicating density variations. The horizontal axis is the rank of each postal code ordered by observed arrivals, while the vertical axis is the number of observed migrants. We only show postal codes with arrivals above five during the observation period. B)—The mean square error of the diaspora model (red) and the gravity model (grey) over 100 runs. C)—Modelled and observed arrivals to Austria. The disc size is proportional to the pre-existing population at the postal code, and the arrivals from our top diasporas are highlighted in different colors following Fig. 2.

To assess the predictive power of the diaspora model, we compare it with the gravity model for the top nationalities arriving in Austria (Fig. 3). To detect how precise both models are, we take samples from the corresponding distributions and estimate the number of arrivals for each destination. To quantify the uncertainty of the models, we repeat the same process of sampling possible results 100 times. The mean square error of the gravity model is 2.85 times bigger than the diaspora model. The gravity model is particularly weak in differentiating nationalities but also for small geographical areas and does not offer a clear metric for predicting arrivals (Supplementary Material C).

**Fig. 4.**
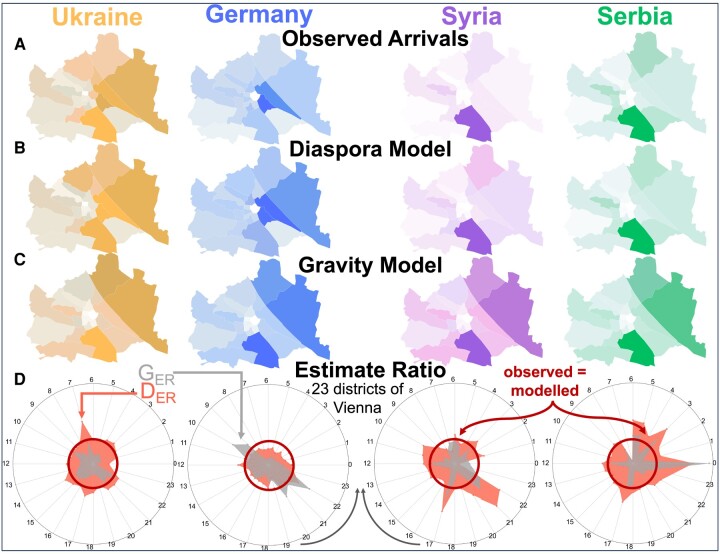
Vienna model results. A)—Heat map of Vienna of the observed arrivals in Austria for the four top diasporas in Austria. B)—Heat map of the diaspora model estimates in Vienna. C)—Heat map of the gravity model estimates in Vienna. D)—Spider plots of the top four diasporas in Austria, where each section is one of Vienna’s 23 districts. The ratio between the modeled and the observed arrivals—estimate ratio—are displayed for each district for both the gravity model in gray ($G_{\mathrm{ER}}$) and the diaspora model in red ($D_{\mathrm{ER}}$). The inner circle (dark red) is when the observed and the modeled arrivals are equal. When the polygons are smaller than the circle, the model underestimates the number of migrants but overestimates that number when it is bigger.

Migrants are more likely to move to destinations with a significant diaspora, not necessarily places with a large population. However, places with a considerable diaspora also tend to have a large population, so the gravity model works relatively well in those limited cases. Nevertheless, the gravity model fails to capture details at small geographical scales. We compare the results of our model with a gravity model at the neighborhood level. Vienna is divided into 23 districts (or “Bezirke”). They are numbered “outwards”, so the first district is the city center, and the 20th–23rd districts are suburbs. The districts tend to be highly heterogeneous regarding demographic and income compositions. Vienna’s 10th district is known for being highly multicultural, attracting nearly 8.3 times more people from Serbia than from Germany. In contrast, the 7th district (known for being the trendy shopping district) attracted 1.4 times more people from Germany than Serbia (Fig. 4). There are many reasons why Germans are more likely to move to one neighborhood in Vienna and Serbians to a different one, but similar reasons have applied to previous migrants. Similar patterns are observed within other countries of origin and city neighborhoods.

### Migration flows in the United States of America

**Fig. 5.**
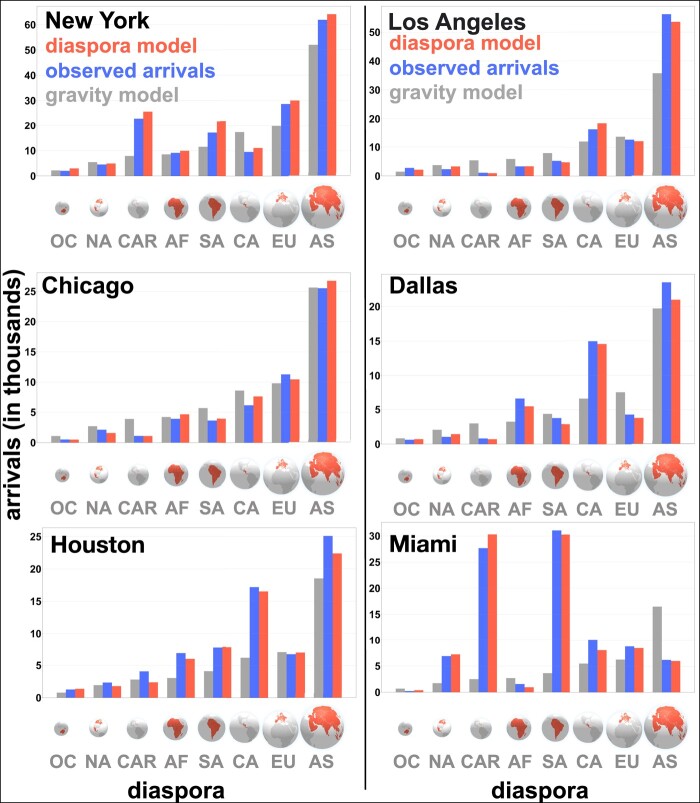
Results of the arrival flows of the top metropolitan areas in the United States of America: New York, Los Angeles, Chicago, Dallas, Houston, and Miami. We plot the observed flows (blue), the diaspora model estimates (red), and the gravity model estimates (grey) for eight estimated diasporas: Oceania (OC), North America (NA), Caribbean Islands (CAR), Africa (AF), South America (SA), Central America (CA), Europe (EU) and Asia (AS). The diasporas are ranked according to their total arrival flow in the United States of America in 2019. The smallest diaspora is from Oceania, with around 1,10,000 individuals, while the largest is from Asia, with more than 25 million migrants.

Homophilic migration is also observed in the United States of America. International migrants to the United States of America form a stable process with repeated geographical patterns. Based on the number of arrivals from different parts of the world to a metropolitan area in the United States of America, we estimate the arrivals in the next year (more details in the Methods section). The results are also repeated patterns. People from South America, for example, are four times more likely to move to Miami than to Houston. However, the opposite applies to people from Central America, who move more frequently to Houston instead (Fig. 5). This phenomenon has persisted for years, although both metropolitan areas have roughly the same population and are at a similar distance to both origins.

Migrants do not necessarily choose large or closer cities as their destination. For example, people from Africa are more likely to move to Washington, D.C., than to New York City, even though New York City has three times more population. On the contrary, people from Europe are two times more likely to move to New York City than to Washington, D.C. The diaspora model can distinguish previous migrant populations and their assortativity, which allows us to estimate the inflow of migrants with high precision.

We test the diaspora model and compare it with the gravity model. For example, the diaspora model predicts 91,694 migrants to the Miami metropolitan area from all regions considered, but the gravity model only predicts around 39,492 migrants (Supplementary Material D). However, we observed around 92,500 migrants. This underestimation from the gravity model is persistent in large metropolitan areas such as Washington, D.C., New York City, Houston, and San Francisco. In those cities, there is already a significant migrant population, indicating either that factors will repeat for new migrations, or with a diaspora acting as a pulling force.

## Discussion

Understanding population dynamics and the impact of the arriving migrants into a country is crucial to planning the provision of services and integrating people into the hosting society. Due to conflict and a demographic expansion in the countries of origin, combined with the decline in the birth rates in other parts, the share of international migrants will keep increasing in the upcoming decades (9). Thus, accurately modeling migration flows will enhance how migration is managed across countries.

Unlike other migration models, which focus only on where migrants move, we distinguish two components of migration: intensity (the number of migrants) and assortativity (their destination) once they have decided to move to a new country. For some country of origin, the intensity is modeled as a homogeneous process, meaning that a similar number of arrivals is expected daily. Although there are minor fluctuations (fewer arrivals during the weekend, for example), and there may be some seasonality and other fluctuations, the overall trend shows that the number of arrivals is roughly stable. We show that the size of the diaspora in the country can approximate the intensity rate. Then, the assortativity captures how destinations with big diasporas attract more people from the same country of origin. Our model uses the diaspora’s size as the only input and explains migration at small geographical units, such as neighborhoods. It is usually difficult to know why many people have moved to a place, but those reasons persist over time and may apply to others. The principle is that most reasons for moving to a new neighborhood remain and keep attracting similar people. Adding the arrivals at the neighborhood level gives the estimate at the city level, which also may be combined to obtain an estimate at the state or country level. Thus, diaspora size can be a unique factor in predicting where migrants will move. The two components can be analyzed separately. By explicitly modeling the intensity of migration, the result could be combined with a gravity or radiation type of assortativity. Further research can be expanded by integrating the modeled intensity with other migration models and other types of data, such as points of interest or job offers.

**Fig. 6.**
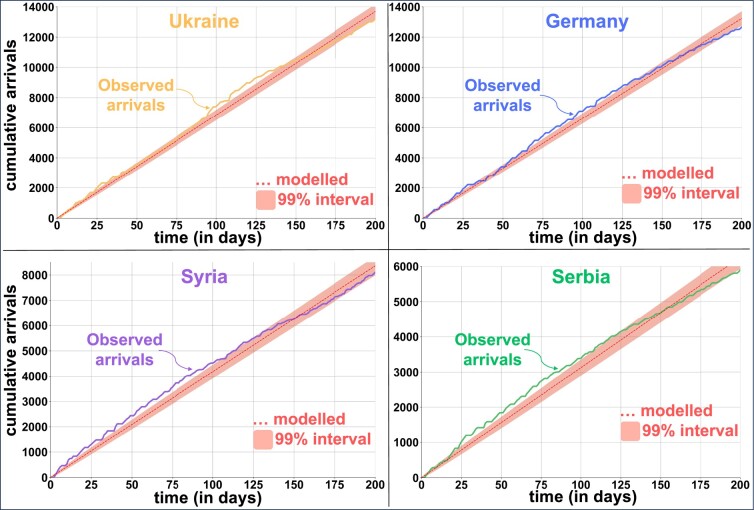
Estimating the pull rate ($\lambda_{i}$). The intensity of migration for country *i* gives the daily arrival rate for that country $\lambda_{i}$. For *t* days, the expected number of arrivals is $\lambda_{i}t$, plotted as a dashed line for each country. For a sufficiently large rate and number of days, a Normal approximation gives a 99% confidence interval, plotted for each country as the shaded triangle (with θ=4). The observed number of arrivals falls within the shaded triangle, so we do not reject a constant arrival rate for those countries of origin.

In considering the applicability of our model, it is important to recognize its limitations. Our model does not predict migration shocks resulting, for example, from a crisis, as they fall outside the scope of its predictive abilities. However, dividing migration into intensity and assortativity enables modeling migration shocks by altering the intensity of arrivals. In the case of a shock elsewhere, the migration intensity will shift to an unknown value, but the assortativity will still explain where most people will be inclined to move. Nevertheless, when the arrival rate of migrants going through a crisis attains stable features—after a certain period, for example, in Syria and Ukraine—we can alter the intensity of arrivals and predict their expected assortativity. Our model may not be applicable in the early stages of migration when no diasporas are present in the corresponding region. Similarly, when the number of migrants is too large, some other elements may gain prominence, such as return migration, a reduction of the number of people who are likely to move, or stricter policies for settling. For example, between 2005 and 2015, more people moved from the United States of America to Mexico than in the other direction (67). Thus, when the diaspora becomes too large, the model may be less applicable. Furthermore, migration might be subject to seasonal effects, such as a slight decay in the inflow of individuals during the summer (Fig. 6). Further research leveraging longer observation periods could verify and quantify seasonal trends. Additionally, we do not account for modes of travel or socio-economic characteristics of migrants that influence their decisions, for example, in the migration of the South American and Central American diasporas to Houston and Miami (Fig. 5).

Regarding reproducibility, our model’s applicability extends beyond the confines of specific regions or time-frames, yet certain constraints must be acknowledged. While we have primarily focused on Austria and the United States of America due to the challenge of obtaining flow data for most countries, assortative data are easier to find for many countries. The assortativity can be modeled by leveraging international stock data (for instance, data provided by the UN (68)). As for the pull rate for destination countries *ρ*, many reasons could influence incoming flows for a specific diaspora size, including migration policies, cultural and historical connections, social welfare systems, political stability, and potential economic gain.

People arriving from different countries go to specific neighborhoods, so segregation is one of the unintended consequences of this process. Migrants do not necessarily seek to be surrounded by their diaspora, but frequently they are. The diaspora model highlights one of the biggest challenges of migration. Minorities concentrate, fostering fewer interactions with others. This process has important implications, as integrating foreigners into national life is complicated when migrants form segregated communities. Our model helps illuminate the mechanisms when migrants choose their destination and guide policies for designing more inclusive and integrated societies. Governments and international organizations must support local authorities and implement strategies to improve migrant inclusion in urban areas (69).

## Methods

### Data

Arrivals and diaspora size for Austria were provided by the Federal Ministry of Interior of Austria, Bundesministerium für Inneres “BMI”. The data include all individuals in Austria who register their residence through the mandatory registration form called the “Meldezettel”. We also have data on asylum seekers of all stages, whether seeking asylum, approved or rejected, and displaced migrants, for example, due to the Russian invasion of Ukraine. Our data do not cover short-term visitors, (for example tourists) who are not obligated to register. In addition, we cannot quantify or detect undocumented migrants. Thus, they are not included in our analysis.

Data for the United States of America were obtained from the American Community Survey—Census data (70). The data contain the resident population and an estimate of the number of arrivals to each metropolitan area (391 areas in total). The data include the person’s residence the year before the survey but not previous years, so repeat migration is not observed. International arrivals are grouped into eight categories: four in America (North America, Central America, South America and the Caribbean) and Africa, Asia, Europe, and Oceania. Data are available in yearly intervals aggregated in 5-year periods, from 2009–2013 to the 2015–2019 surveys.

### Constructing a diaspora model for the migration

We decompose the migration process with two hierarchical components: the migration intensity (related to the number of migrants from some country) and the assortativity (related to the destination). We use a two-step hierarchical model to consider the two steps separately. This method is frequently used in other domains, where a random variable is modeled by considering ordered steps (60).

#### Modeling the migration intensity

We start with the number of migrants and assume it follows a Poisson distribution such that:


(2) $$M_{i}(t)∼\text{Pois}(\lambda_{i}t),$$

where $M_{i}(t)$ is the flow of migrants for a period of *t* days, from the country of origin *i*, where i=1,2,…,μ and with a daily rate $\lambda_{i}$. The Poisson distribution is frequently used to model a variable that results from a counting process, such as migrants (64). The distribution depends on a single rate, and it is used to ignore short-term fluctuations and look only at the more general pattern. The expected number of migrants until day *t* is $\lambda_{i}t$, an expression for the cumulative number of arrivals from country *i*.

First, we test if a uniform rate works during *n* days to estimate the arrival rate from different countries. The error term for day *i* gives $e_{i}(t)=M_{i}(t)-\lambda_{i}t$. The sum of square errors over the observed days gives $f(\lambda_{i})=\sum_{t=0}^{n}e_{i}{\left(t\right)}^{2}$. By setting $f^{\prime }(\lambda_{i})=0$ we obtain that


(3) $$\lambda_{i}^{⋆}=\frac{6\sum_{t=0}^{n}tM_{i}(t)}{n(n+1)(2n+1)}.$$

Since f(λ) is a continuous function and $f^{\prime \prime }(\lambda_{i})=n(n+1)(2n+1)/3>0$, then the value $\lambda_{i}^{⋆}$ minimizes the error. For a sufficiently large number of days and arrival rate, a Normal approximation to the Poisson distribution may be used to obtain the corresponding confidence interval, given by $\left[\lambda_{i}t-\theta \sqrt{\lambda_{i}t},\lambda_{i}t+\theta \sqrt{\lambda_{i}t}\right]$. For a lower rate (or for fewer days), a Monte Carlo method may also be used to obtain plausible departures. For the top migrant countries to Austria, the estimated arrivals are approximated by the constant rate (Fig. 6).

There are some fluctuations in the daily number of arrivals. For example, very few people arrive during the weekends. However, a fixed arrival rate works well for modeling the daily arrival of migrants and enables us to ignore minor fluctuations. Equation 3 may be used to estimate the daily arrival rate for migrants from different countries. Although Eq. 3 estimates the arrival rate, we then aim to approximate the rate based on the size of the country’s diaspora. The motivation is to model whether a bigger diaspora results in more arrivals (as observed in Fig. 2). We take the data for all countries, considering that


(4) $$M_{i}(t)=\rho R_{i}t,$$

where $R_{i}$ is the size of the diaspora from country *i*, and ρ>0 is a fixed pull rate for all countries of origin and depends only on the arrivals and existing diasporas at the destination. Thus, we assume that for a given country, the flow depends on the size of the diaspora $R_{i}$ and some fixed pull rate *ρ*. Following the same logic, we obtain the value of *ρ* by minimizing the errors such that the error $e_{i}=M_{i}-\rho R_{i}t$. The sum of the squared errors over the observed days gives $g(\rho )=\sum_{i}e_{i}^{2}$ By setting $g^{\prime }=0$, we obtain that


(5) $$\rho^{⋆}=\frac{\sum_{i=1}^{\mu }M_{i}R_{i}}{t\sum_{i=1}^{\mu }R_{i}^{2}},$$

an estimate for the pull rate that depends on the arrivals and diaspora of all countries. The second derivative of *g* is $2\sum_{i=1}^{\mu }R_{i}^{2}t^{2}>0$, so the value of $\rho^{⋆}$ minimizes the sum of the squared differences. Equation 5 depends on the size of the diaspora and the arrivals over *μ* countries, unlike Eq. 3, which depends on the daily arrivals for a single country.

In the case of Austria, we obtain that $\rho_{\mathrm{Aus}}=3.29\times 10^{-4}$. Then, we can use the diaspora size and the estimated pull rate to express the arrival rate for country *i* as


(6) $$\lambda_{i}=\rho R_{i}.$$

Equation 6 gives the arrival rate from county *i* considering the diaspora size of that country and a pull rate *ρ* that applies to all countries equally. This method overestimates arrivals from some countries (for example, Serbia and Turkey) and underestimates others (for example, Romania and Germany) and, in general, results in higher error than Eq. 3 (Supplementary Material C). However, it gives an alternative expression to estimate rates that do not depend on the data for the daily arrivals to that country. The obtained value of $\rho_{\mathrm{Aus}}=3.29\times 10^{-4}$ reflects that one person is expected to arrive daily for every 3,031 individuals from any diaspora. For example, in Austria, there are around 42,580 people from Poland, so 14 people are expected each day, or 2,800 people during the 200 days of observation. The observed number of arrivals from Poland during that period was 2,787 migrants (Supplementary Material A).

#### Modeling the migration assortativity

Once the number of arrivals is known, we model their conditional destination in that country (31, 71, 72). We assume that once $M_{i}(t)=m$ persons arrive, they decide to reside in a particular location *j*—for example, a city in the destination country—depending on the size of the diaspora in the location *j*. Thus, we assume that the probability of a person from country *i* moving to location *j* follows:


$$\pi_{ij}=R_{ij}/R_{i},$$

where $R_{ij}$ is the diaspora from country *i* in destination *j*. The diaspora is such that $R_{i}=\sum_{j}R_{ij}$ is the overall size of the diaspora from country *i*. For example, if location *j* has 10% of the diaspora from *i*, we assume that the probability that a migrant moves to *j* among the *ν* destinations is also 10%. Destinations with bigger diaspora attract more migrants. The process is modeled as a multinomial distribution:


$$M_{ij}(t)|M_{i}(t)=m∼\text{Mult}(m,{\bar{\pi }}_{i}),$$

where $M_{ij}(t)$ are the arrivals from *i* in location *j* and ${\bar{\pi }}_{i}=(\pi_{i1},\pi_{i2},\ldots ,$$\pi_{i\nu })$ is the vector with entries $\pi_{ij}$ corresponding to the probability of choosing *j* as their destination, where $\sum_{k}\pi_{ik}=1$. A multinomial distribution, conditional on a Poisson distribution, also follows a Poisson distribution with combined rates of arrivals and success (64). Therefore, arrivals from country *i* to location *j* follow $M_{ij}(t)∼\text{Pois}(\pi_{ij}\lambda_{i}t)$.

If the daily rate of arrivals is known, then Eq. 3 gives an estimate of the arrivals at a granular level. If the diaspora size from other countries is known, then Eq. 6 gives an estimate that depends only on the diaspora size from country *i* in destination *j*. Combining both, arrivals from country *i* to location *j* follow


(7) $$M_{ij}(t)∼\text{Pois}(R_{ij}\lambda t).$$

Other migration models focus only on the assortativity of individuals, meaning that they only differentiate the likelihood of moving between destinations but ignore the number of people moving. However, migration intensity is more relevant than assortativity. Modeling how many people will arrive in a country should be the first explicit component of migration models. Arrivals can be modeled with a constant rate so that they can be predicted within a reasonable period. Further, the arrival rate is strongly linked to their diaspora size. We estimate how many people will move to a country if we observe how many people already live there. The second element, related to assortativity, has been captured by size, job offerings, and others. Yet, diaspora size explains assortativity more accurately than population size and with smaller geographical units than metropolitan areas.

### Inferring diaspora size and subsequent migrations

In the case of international migration to metropolitan areas in the United States of America, census data estimate the yearly arrivals between 2009 and 2019 (70). It is disaggregated for eight regions of origin (Africa, Asia, the Caribbean, Central America, Europe, Northern America, Oceania, and South America). It gives the number of arrivals to 391 metropolitan areas and the countryside. We use the number of arrivals in 1 year to estimate arrivals in the subsequent one. Let $A_{ij}(t)$ be the number of arrivals from region *i* to metropolitan area *j*. We assume that the arrivals in that year result from some fixed pull rate *λ* and an unknown diaspora size $D_{ij}$. Then, the expected number of arrivals for the next year follows a Poisson distribution with rate $\lambda D_{ij}=A_{ij}$ for some value of λ>0. Thus, the expected number of arrivals in 1 year is the observed number during the previous year.

We compare the estimated number of arrivals $E_{ij}$ to the observed number $A_{ij}$ in 2019. At the regional level, for example, the model gives that 131,478 arrivals are expected from Africa, and between 130,767 and 132,188 arrivals are expected. The observed number of migrants from Africa in 2019 was 131,943. For other regions, the estimated inflow is within a 3.5% difference from the observed number of arrivals, except for the case of South America. The yearly number of migrants from South America to the United States of America has nearly doubled in 7 years. This increasing intensity in the annual number of migrants would be better captured considering the observed trend (more details in Supplementary Material D).

### Constructing a gravity model for the migration

Other migration models, such as gravity or radiation, have many drawbacks. Often, they produce a deterministic number of arrivals to each destination, and frequently, they focus only on the assortativity of individuals, meaning that they only differentiate the likelihood of moving between destinations but ignore the number of people moving. However, migration intensity is more relevant than assortativity. Modeling how many people will arrive in a country should be the first explicit component of migration models, which is often overlooked. In most expressions of the gravity or the radiation model, only the assortativity is considered (27, 28, 33, 34, 38). The second element, related to assortativity, has been captured by size, job offerings, and others. For example, the gravity model can be expressed as follows. Instead of taking the diaspora size, we consider the total population of the destination and express the arrivals $G_{ij}$ from *i* to *j* as


$$G_{ij}(t)|M_{i}(t)=m∼\text{Mult}(m,{\bar{\pi }}^{\left(G\right)}),$$

where ${\bar{\pi }}^{\left(G\right)}=(P_{1}/P,P_{2}/P,\ldots ,P_{\nu }/P)$ is the relative size of each city at the destination, and it is the same for all counties of origin. The diaspora size explains assortativity more accurately and with smaller geographical units than metropolitan areas. Because large cities tend to have a high diaspora share, the gravity model works relatively well to capture a general trend. However, the gravity model fails at smaller geographical units. According to the gravity model, two neighborhoods of similar size are equally attractive to migrants, but this is never the case. Ethnic neighborhoods, such as “Chinatowns”, are part of the cultural landscape in most cities.

## Supplementary Material

pgae178_Supplementary_Data

## Data Availability

The raw and processed data are not available due to privacy laws. The Federal Ministry of the Interior of Austria safeguarded the dataset and made it accessible to our research institution under strict data protection regulations. Researchers must find individual agreements with the Federal Ministry of the Interior of Austria to access this data. Data for the United States of America can be accessed through the American Community Survey website (www.census.gov/programs-surveys/acs).

## Previous Presentation

These results were previously presented at the Conference on Complex Systems CCS2023, 16 October 2023.

## Preprints

A preprint of this article is published at arXiv:2309.03070.
