# Supplementary material for: The diaspora model for human migration
Source: PNAS Nexus. 2024 May 21;3(5):pgae178. doi: 10.1093/pnasnexus/pgae178 (PMC11107377; doi:10.1093/pnasnexus/pgae178)
Supplement: pgae178_Supplementary_Data [file pgae178_supplementary_data.zip › PNASNEXUS-PNASNEXUS-2023-01187RR-s08.pdf]

PNAS nexus

Supporting Information for

The diaspora model for human migration

Rafael Prieto-Curiel, Ola Ali, Elma Dervic, Fariba Karimi, Elisa Omodei, Rainer Stütz, Georg Heiler, Yurij Holovatch

Rafael Prieto-Curiel
E-mail: prieto-curiel@csh.ac.at

This PDF file includes:

Figs. S1 to S3
Tables S1 to S7
SI References

A. Data description and observation. The data corresponding to arrivals to Austria is provided in the **Complex Effects of Migration Patterns on Supply Capacities** project. Access to the data is restricted for security and privacy reasons, and only authorised users can view the data. For each person, the data includes their country of origin and the neighbourhood in which they have a registered residence. The data gives the location of the diaspora for each country at the postal code level. Data before November 26, 2022 (referred to as "December" in the manuscript) does not identify each arrival date. It gives the age, gender, residential status, and country of origin of $R = 1,466,113$ migrants in Austria. For 200 days, the majority of arrivals to the country were captured at the moment when a person registered their residence through a registration form known as the "Meldezettel" with the Bundesministerium für Inneres (Federal Ministry of the Interior) when they apply for some form of residence permit in the country. Visits planned for shorter periods (tourism) do not require registering and are not counted.

Legally, migrants in Austria are classified according to their residence status. For example, migrants who plan to stay in the country for less than six months are classified as foreigners, but if they stay longer, they are classified as settled migrants with a residence permit. On the other hand, refugees fall under different classifications depending on their stage, for example, seeking asylum, approved or rejected. As of 14 June 2023, refugees cover only 13.72% in our data (Table S1). We apply the same analysis to all classifications.

Migration Status	Percentage
Settled migrant with a residence permit	54.30 %
Foreigner	31.76%
Entitled to asylum	10.78%
Eligible for subsidiary protection	1.70%
Approved asylum seeker	0.63%
Displaced person	0.53%
Humanitarian residence permit	0.08%
Asylum seeker	0.01%
Other classifications	0.21%

Table S1. Migrants residential status as of 14 June 2023

The data contains information describing the nationality and residence of 1.46 million people from 192 countries. As of November 26, 2022, Austria has 1,542,349 registered migrants from 192 countries. Around 95% disclosed the main addresses and are considered here. We analyse the arrivals for 263 days divided into two parts: 200 days to train and 63 days to test. Within the period of analysis that considers 200 days, there were $A = 111,244$ arrivals to the country, mainly from Ukraine, Romania, Germany and Syria. As of 14 June 2023 (after 200 days), around 75% of arrivals are from 15 countries (Table S2).

Country of origin	Diaspora	Arrivals	Arrivals %
Ukraine	76,577	12,054	10.84%
Romania	102,314	11,065	9.95%
Germany	115,913	10,064	9.05%
Syria	82,746	8,030	7.22%
Hungary	65,269	7,794	7.01%
Croatia	89,296	5,177	4.65%
Turkey	135,205	4,926	4.43%
Serbia	145,348	4,536	4.08%
Bosnia and Herzegovina	109,478	3,462	3.11%
Afghanistan	52,821	3,086	2.77%
Bulgaria	27,474	2,934	2.64%
Poland	42,580	2,787	2.51%
Slovakia	32,363	2,650	2.38%
Russia	41,013	2,508	2.25%
Italy	21,581	2,264	2.04%
Other countries	326,135	27,907	25.09%
**Total**	1,466,113	111,244	_

Table S2. Top countries of origin in Austria, in descending order of arrivals within the observation period of 200 days. Only migrants with registered main addresses. We list countries with arrivals percentages above 2%. The diaspora is the country's pre-existing population size - before November 26, 2022.

We test whether a uniform daily arrival explains the observed number of migrants from the top countries of origin. A uniform daily arrival is not rejected for the top 12 countries. The observed arrivals fall within the modelled intervals (Figure S1). We also test whether postal codes with a larger diaspora attracted more migrants.

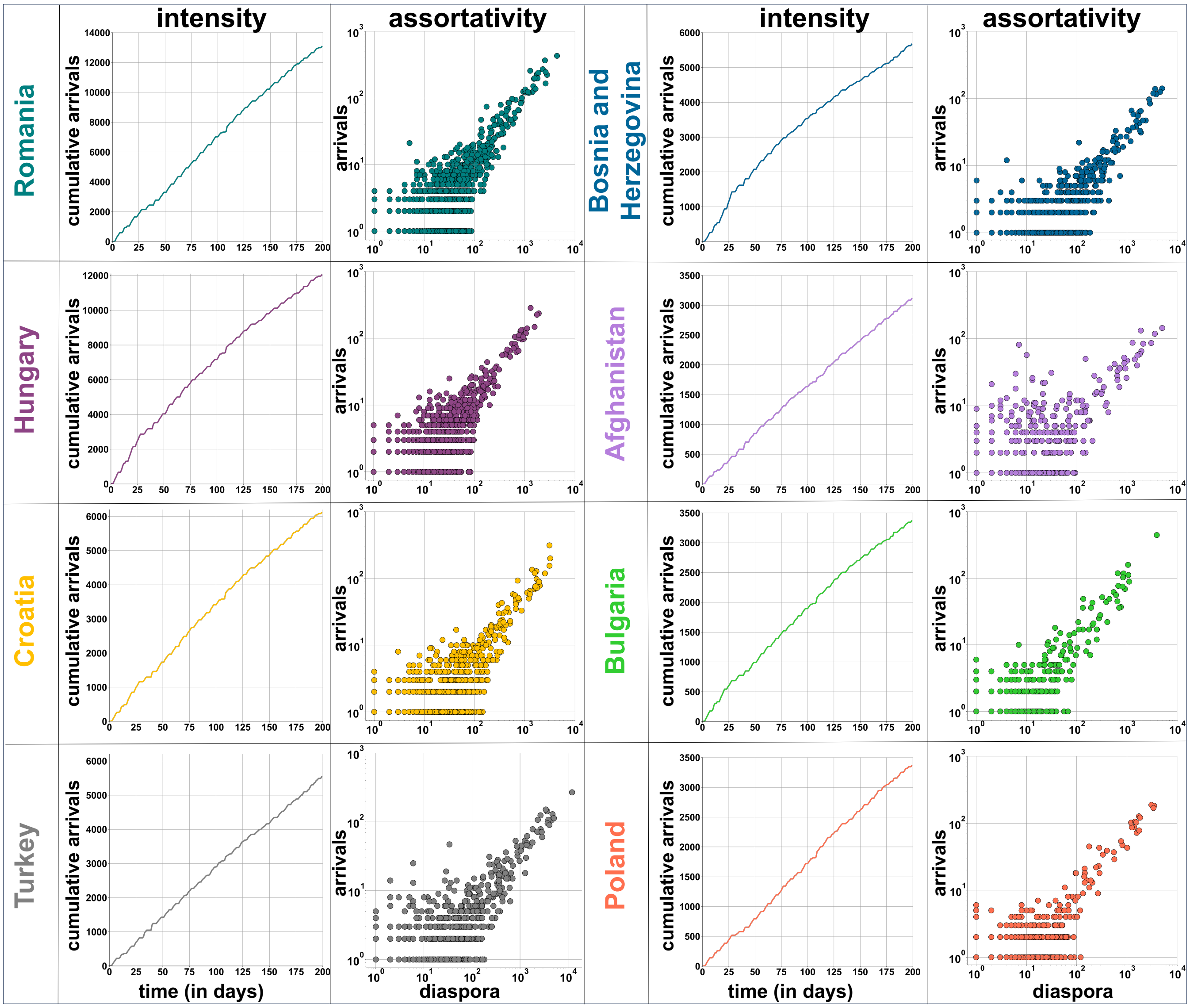

Fig. S1. Intensity and assortativity of the top Diasporas. The intensity of Arrivals in the 200 days of observation of the top eight diasporas in Austria (left). The assortativity of the arrivals of the top eight diasporas (right).

B. Modelling intensity and assortativity. Modelling intensity and assortativity separately enables the disentangling of the process with a minimal set of parameters. First, let $M_i(t)$ be the number of arrivals from country i since time $t = 0$. We assume that $M_i(t) \sim \text{Pois}(\lambda_i t)$, so the expected number of arrivals during t days is $\lambda_i t$. The Poisson distribution is frequently used to model discrete events (such as the number of arrivals) since it allows overlooking small perturbations or fluctuations and focuses on the more general picture, the daily arrivals. It depends on a single parameter, λ_i, known as the (daily) rate, which is the expected number of arrivals per day.

Once a person decides to move to some country, they decide on a specific location, which can be as general as states or provinces or as particular as neighbourhoods. The person chooses location j with probability π_{ij}. The destination, conditional on observing m arrivals, can be considered a Multinomial distribution with ν options. The vector $\bar{\pi}_i = (\pi_{i1}, \pi_{i2}, \dots, \pi_{i\nu})$ captures the destination preferences for people from origin i. In particular, the decision of moving to destination k, with $k \in 1, 2, \dots, \nu$, is a Binomial distribution (with a probability of success π_{ij} and with a probability of failure $1 - \pi_{ij}$). Thus, the number of arrivals to destination k, conditional on observing m arrivals, is given by

$$M_{ik}(t)|M_i(t) = m \sim \text{Bin}(m, \pi_{ik}). \quad [1]$$

It is possible to show that a Binomial distribution, conditional on a Poisson distribution, is also a Poisson distribution (1). It has a rate $\lambda_i \pi_{ik}$, which is the same rate but discounted by the probability π_{ik}. Thus, arrivals to destination k are $M_{ik}(t) \sim \text{Pois}(\lambda_i \pi_{ik} t)$. Modelling intensity and assortativity separately enables the disentangling of the process with a minimal set of parameters.

The assortativity of the diaspora model of migration estimates that people from i move to location j with probability $\pi_{ij} = R_{ij}/R_i$, where R_{ij} is the diaspora size of country i in location j, and $R_i = \sum_j R_{ij}$ is the total diaspora. After some period, t, the new diaspora will have size $R'_{ij} = R_{ij}(1 + B - D + I - O + \lambda)$, where B is the birth rate, D is the death rate, I is the inflow due to internal movements, O is the outflow due to internal movements, and λ corresponds to the new arrivals. Assuming that the impact of internal migration of the diaspora is negligible (meaning that $I \approx O$), we get that $R'_{ij} = R_{ij}(1 + B - D + \lambda)$. Further, assuming that the birth and death rates are similar for all the diasporas (so $B \approx D$), we get that $R'_i = \sum_j R'_{ij} = R_i(1 + \lambda)$, so the total diaspora also changes size due to the arrival of people. Then, the assortativity impact, after some period is $\pi'_{ij} = R'_{ij}/R'_i = \pi_{ij}$, so it remains unchanged. Thus, the model conserves the distribution of the diaspora across destinations after the arrival of people is considered.

C. Model comparison. In this section, we compare our model with the gravity model. The gravity model is one of the most prominent ways in which social mobility is analysed. The gravity model captures the impact of size at the origin and destination countries and their distance (2–6). Gravity has been used, for example, to model trade between countries and cultural distances or frictions between distinct locations (2, 3, 7). The gravity model, however, does not quantify the intensity of migration but gives only a description of the assortativity. One of the most significant drawbacks of the gravity model is that it does not consider any temporal dimension, so it only ranks destinations depending on their size. Unfortunately, the gravity model does not provide the expected arrivals of migrants or an analogy to our diaspora pull rate; thus, we do not include it in the intensity error calculations.

C.1. Intensity. To assess the error in the expected arrivals of migrants, we use the 200 days of observations to construct a daily pull rate for every country λ_i (Equation 3) and predict the arrivals in the next nine weeks (63 days since 14 June 2023). We choose to have a time window in weeks instead of months because migration patterns and data registration go through a weekly cycle. We use two different approaches to calculating the intensity of migrants. The first approach is based on the arrival rate of country i to the destination country. It is calculated using the cumulative daily arrival data. This approach is data intensive, requiring daily resolution of the historical diaspora arrival rate. It is expressed as λ_i (shown in Figure 6 and Equation 3 in the manuscript). This approach can predict the observed arrivals with a margin of ± 0.17 arrival per country per day (Table S3).

In the second approach, the arrival rate of any country to the destination is calculated by fitting the total arrivals from all countries of origin within time widow t. It uses equation 5 (in the manuscript). This approach is less data-intensive as it only requires the diaspora sizes at the beginning of the observation and the arrivals at a time $(t_0 + t)$. This calculation is expressed by ρ, shown in Figure 2 A (Left in the manuscript). We estimate the arrivals for all countries and find that using this method, our model can predict the observed arrivals with a margin of ± 0.32 arrival per country per day. Thus, using ρ_{Aus}, we get almost twice the error. However, we can rely on fewer data points.

Country	λ_i	Arrivals	Arr(λ_i)	Arr(ρ_{Aus})	Err(λ_i)	Err(ρ_{Aus})
Ukraine	68.48	3,106	4,314	1,842	1,459,264	1,597,696
Hungary	65.46	3,264	4,124	1,519	739,600	3,045,025
Syria	41.79	3,309	2,633	1,887	456,976	2,022,084
Germany	66.18	3,579	4,170	2,618	349,281	923,521
Romania	67.44	3,713	4,249	2,356	287,296	1,841,449
Slovakia	26.59	1,180	1,675	728	245,025	204,304
Bosnia and Herzegovina	31.05	1,462	1,956	2,347	24,4036	783,225
Serbia	31.25	1,561	1,969	3,115	166,464	2,414,916
Bulgaria	17.83	796	1,123	632	106,929	26,896
Italy	17.30	833	1,090	496	66,049	113,569
All Countries	_	39,360	44,559	32,777	4,384,765	15,312,703
$\sqrt{\sum (E_r)^2}$	_	_	_	_	± 0.17	± 0.32

Table S3. Top 10 errors comparisons of the observed and modelled arrivals where Arrivals are the observed arrivals, λ_i is the daily pull rate of every country, ρ_{Aus} is the daily pull rate of Austria, Arr(λ_i) are the modelled arrivals using λ_i, Arr(ρ_{Aus}) are the modelled arrivals using ρ_{Aus}, Er(λ_i) and Er(ρ_{Aus}) are the squared error of Arr(λ_i) and Arr(ρ_{Aus}) respectively. The errors are ranked according to the squared error of λ_i, expressed as Er(λ_i), in descending order for nine weeks (63 days). $\sqrt{\sum (\text{Er})^2}$ is the square root of the sum of the squared error averaged over 63 days of observation and 192 countries.

C.2. Assortativity. For a fixed period, a country of origin i and destination j, we have modelled the flow D_{ij} and compared it to the observed flow M_{ij}. We compute the mean square error as:

$$\text{Er} = \sum_{i,j} \frac{(D_{ij} - M_{ij})^2}{\mu\nu}, \qquad [2]$$

where μ and ν are all the possible origins and destinations. The mean squared error can be used to compare distinct models, where a smaller error means better performance.

The gravity model assumes that destination j with population P_j attracts population depending on its size, so we consider its assortativity as $\pi_{ij}^g = f(P_j, D_{ij})$ for some function f that takes the size of the destination and the distance between origin and destination. We construct a gravity model $\mathcal{G}$ such that once a person has decided to move to a country, they choose their destination depending on its size. Thus, we also consider that the destination is picked as a Multinomial distribution depending on its size. Formally, we assume that once m people have moved from i, they will move to j depending on its size, so $\pi_{ij}^{\mathcal{G}} = P_j^\alpha / \sum_j P_j^\alpha$, for some parameter $\alpha \geq 0$. We compare the diaspora and gravity models by comparing the mean square error (Figure S2).

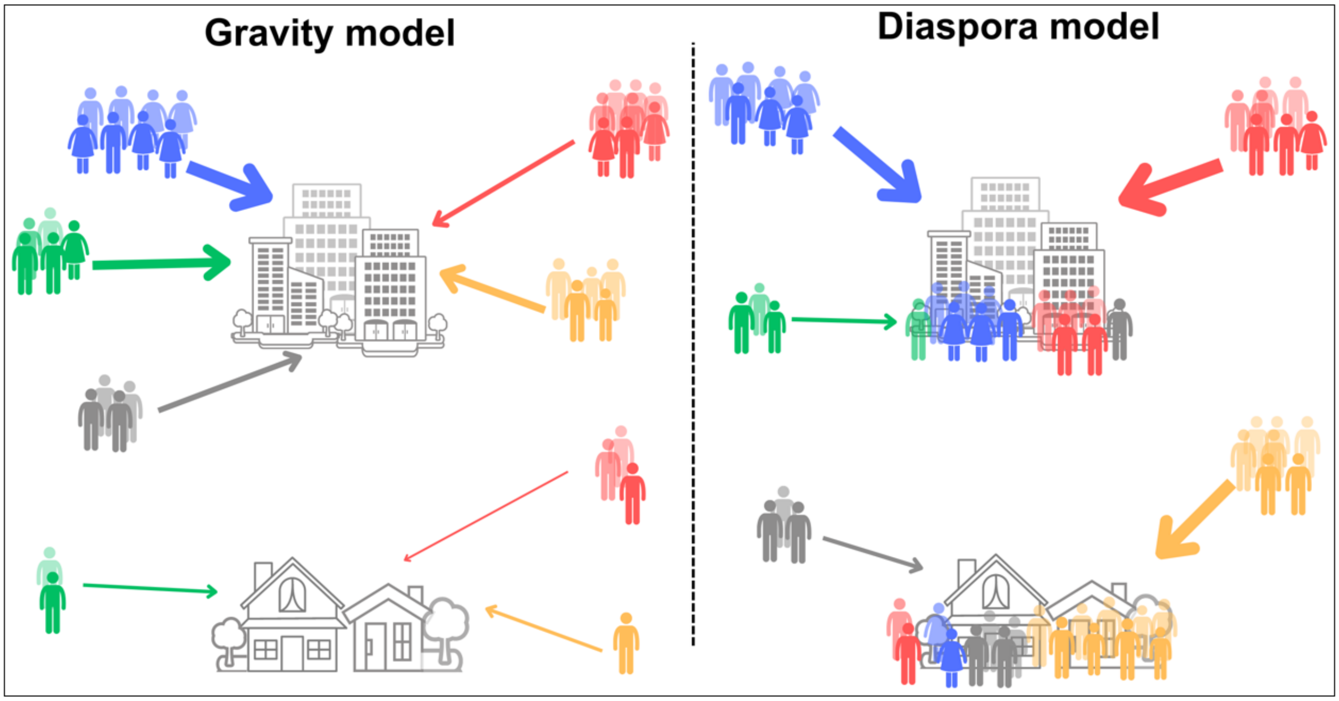

Fig. S2. Gravity and Diaspora model descriptions.. We divide migration into two components: intensity (related to the arrival of individuals) and assortativity (related to where migrants decide to go). The diaspora model of migration uses the size of the pre-existing population of a certain diaspora. The gravity model uses the total pre-existing population without accounting for diasporas and individual differences.

We compare the diaspora and the gravity model by analysing only their assortativity. That is, we assume m arrivals to some destination and distribute them depending on the assortativity of the model considered. We consider the sum of the squared error terms of each model (where a smaller error means that the model describes the assortativity of the arrivals more accurately). The difference between the error terms of the gravity and the diaspora models is enormous, particularly for the countries with the highest number of arrivals. For example, for arrivals from Germany, the gravity model has a squared error of 280.97, but the diaspora model has a squared error of 55.60.

We use the error equation outlined in Equation 2 to calculate the error of each origin country for all destinations. We average the error over 100 simulations for both models and get the average per country (Table S4). In total, there are 192 countries and 2,221 possible destinations.

Country	Diaspora Model square error	Gravity Model square error
Syria	67.39	389.42
Germany	55.60	280.97
Ukraine	56.78	224.34
Morocco	185.32	184.17
Romania	39.36	150.83
Serbia	19.97	123.61
Bulgaria	8.59	91.35
Croatia	21.17	75.07
Hungary	20.59	44.21
Poland	8.19	44.14
All Countries	658.45	1,875.97
Er_r	3.42	9.77

Table S4. Error Comparison between the diaspora and the gravity model. We list the top 10 countries of origin for all postal codes, sorted in descending order according to the gravity model such that the Syria diaspora has the biggest gravity model error and the Poland diaspora has the lowest. Er_r is the mean squared error.

A crucial aspect of migration models is considering different geographic levels. For example, detecting the number of arrivals at the province level is critical since some provisions are frequently managed at that level (such as health or education). However, in smaller units such as cities and neighbourhoods, forecasting the number of migrants plays a critical role. One of the most significant weaknesses of the gravity model is that it cannot predict migration at the neighbourhood level. The gravity model has a squared error of 4,925.15 when we look at the arrivals to the 10th district of Vienna (Favoriten), but the diaspora model has a squared error of 500.76. Results show that the mean square error is 3.42 for the diaspora model but 9.77 for the gravity model. Thus, the average error of each destination for all countries is nearly three times bigger for the gravity model compared to the diaspora model (Table S5).

Postal Code	Diaspora Model squared error	Gravity Model squared error
1100	500.76	4,925.15
6020	393.12	2,288.01
8020	211.23	1,104.15
8055	912.89	950.74
1030	138.67	859.40
1020	110.07	806.55
4880	764.52	788.96
1160	97.56	754.73
1120	161.68	618.12
1200	90.19	488.60
All Postal Codes	7,616.84	21,700.769
Er_r	3.42	9.77

Table S5. Top 10 error comparison between the diaspora and the gravity model per postal code for all countries of origin, sorted in descending order according to the gravity model. The postal code 1100 (Favoriten, 9th district in Vienna) has the biggest gravity model error, and the postal code 1200 (Brigittenau, 20th district in Vienna) has the lowest. Er_r is the mean squared error.

D. International migration to the USA. We conducted our analysis of 387 USA metropolitan areas—Mets. We excluded movements to the countryside, and five metropolitan areas were added to the 2019 census. The census data also limits us to only eight diasporas where the migrants' countries of origin are classified: Asia, Europe, Central America, South America, Africa, the Caribbean Islands, North America, and Oceania. We use the census data from 2013 to 2018 to estimate the arrivals of our selected Mets in 2019.

The gravity model proves insufficient to predict the migration flows with underestimation in big metropolitan areas and overestimation in small metropolitan areas (Figure S3).

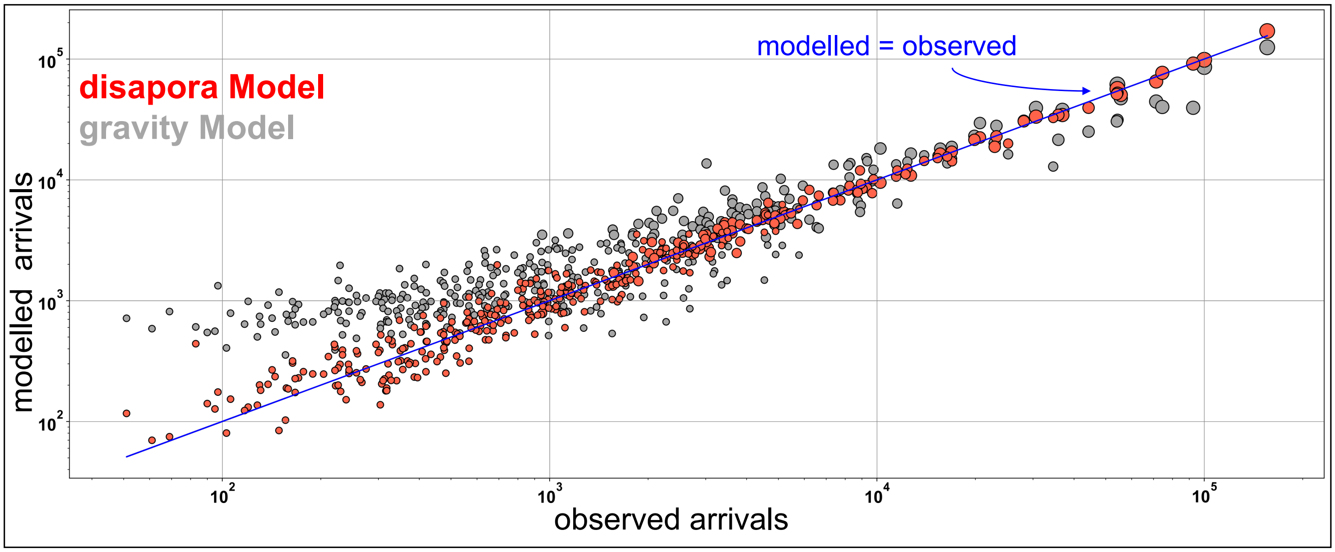

Fig. S3. USA Metropolis. Results of the arrival flows of all the metropolitan areas 387 in the US. We plot the diaspora model estimates (pink), the gravity model estimates (grey) and the observed flows (blue). The sizes of the observations vary depending on the size of Met.

We use the observed total arrivals in both the diaspora and the gravity model, and we model the assortativity according to Met size in the case of the gravity model and according to average diaspora assortativity in the case of the diaspora model (Table S6).

Met Name	Arrivals	Gravity Model	Diaspora Model
New York-Newark-Jersey City	155,722	125,025.22	170,453.85
Los Angeles-Long Beach-Anaheim	99,989	85,882.89	98,584.74
Chicago-Naperville-Elgin	54,275	61,604.83	56,617.11
Dallas-Fort Worth-Arlington	55,590	47,356.62	50,547.42
Houston-The Woodlands-Sugar Land	71,303	44,491.35	65,255.46
Washington-Arlington-Alexandria	74,449	40,127.31	76,847.79
Miami-Fort Lauderdale-Pompano Beach	92,500	39,492.34	91,694.38
Philadelphia-Camden-Wilmington	30,614	39,414.61	33,362.91
Atlanta-Sandy Springs-Alpharetta	36,869	37,973.96	34,408.14
Boston-Cambridge-Newton	54,319	31,350.37	52,575.47
All Mets	1,823,840	1,823,840	1,823,840

Table S6. The observed arrivals of the biggest ten Metropolitan areas in the US in 2019 and their gravity and diaspora model estimates.

We compare the diaspora model results with the observed arrivals and gravity model results, and we show that the mean squared error of the gravity model is 19.3 times bigger than the diaspora model (Table S7).

Met Name	Gravity Model squared error	Diaspora Model squared error
New York-Newark-Jersey City	942,292,280.17	217,027,451.26
Los Angeles-Long Beach-Anaheim	198,982,433.19	1,971,940.83
Chicago-Naperville-Elgin	53,726,416.07	5,485,484.20
Dallas-Fort Worth-Arlington	67,788,545.62	25,427,649.57
Houston-The Woodlands-Sugar Land	718,864,340.74	36,572,692.26
Washington-Arlington-Alexandria	1,177,978,170.36	5,754,213.28
Miami-Fort Lauderdale-Pompano Beach	2,809,811,712.71	649,017.76
Philadelphia-Camden-Wilmington	77,450,653.74	7,556,509.42
Atlanta-Sandy Springs-Alpharetta	1,220,942.69	6,055,836.33
Boston-Cambridge-Newton	527,558,106.12	3,039,895.61
All Mets	9,314,104,678.92	482,227,713.95
Er_r	3,008,431.74	155,758.30

Table S7. The squared error of the gravity and the diaspora model in the ten biggest Metropolitan areas, Er_r is the mean squared error calculated over all diasporas and Mets.

References

1. R Prieto-Curiel, M Quiñones Domínguez, E Lora, N O'Clery, Mobility between Colombian cities is predominantly repeat and return migration. *Comput. Environ. Urban Syst.* **94**, 101774 (2022).
2. JE Anderson, The gravity model, (National Bureau of Economic Research), Technical report (2010).
3. JJ Lewer, H Van den Berg, A gravity model of immigration. *Econ. Lett.* **99**, 164–167 (2008).
4. EG Ravenstein, The laws of migration. *J. Stat. Soc. Lond.* **48**, 167–235 (1885).
5. SA Stouffer, Intervening opportunities: a theory relating mobility and distance. *Am. Sociol. Rev.* **5**, 845–867 (1940).
6. WS Jung, F Wang, HE Stanley, Gravity model in the Korean highway. *EPL (Europhysics Lett.* **81**, 48005 (2008).
7. H Barbosa, et al., Human mobility: Models and applications. *Phys. Reports* **734**, 1–74 (2018).
