# Supplementary figures and images for: The diaspora model for human migration
Source: PNAS Nexus. 2024 May 21;3(5):pgae178. doi: 10.1093/pnasnexus/pgae178 (PMC11107377; doi:10.1093/pnasnexus/pgae178)

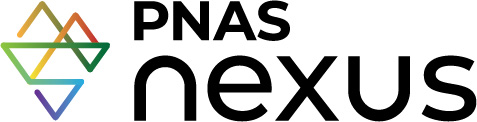

Supplement: pgae178_Supplementary_Data [file pgae178_supplementary_data.zip › PNASNEXUS-PNASNEXUS-2023-01187RR-s05.jpg]

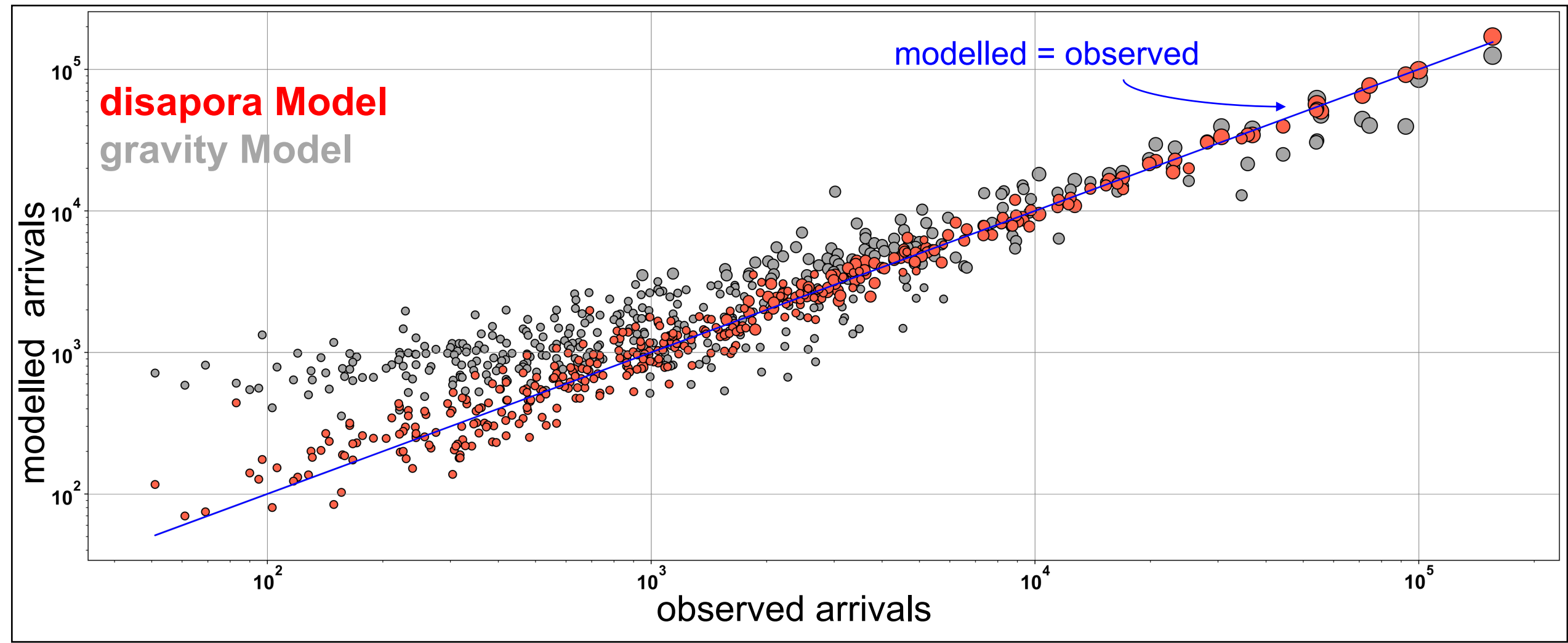

Supplement: pgae178_Supplementary_Data [file pgae178_supplementary_data.zip › PNASNEXUS-PNASNEXUS-2023-01187RR-s06.pdf]

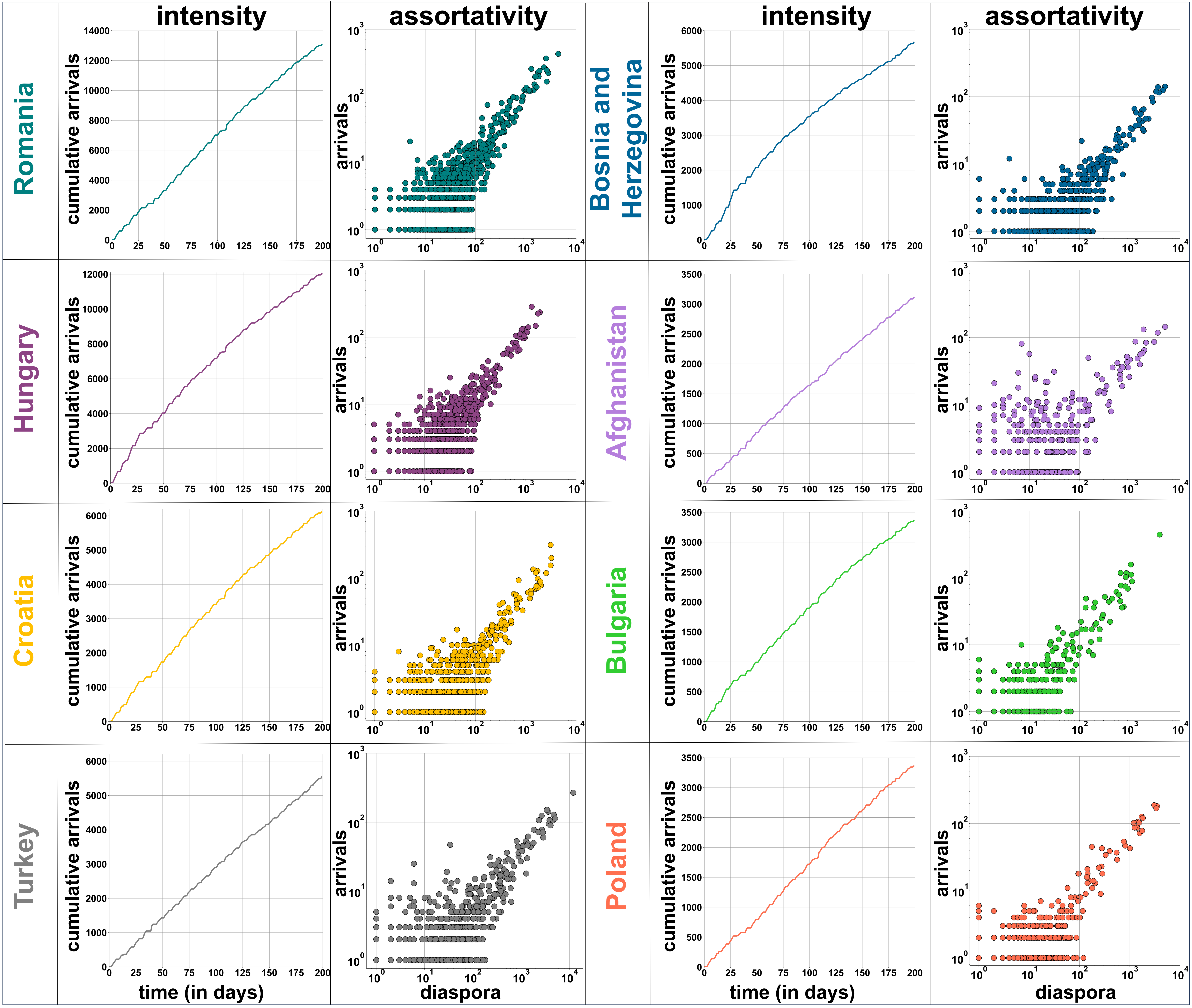

Supplement: pgae178_Supplementary_Data [file pgae178_supplementary_data.zip › PNASNEXUS-PNASNEXUS-2023-01187RR-s07.pdf]

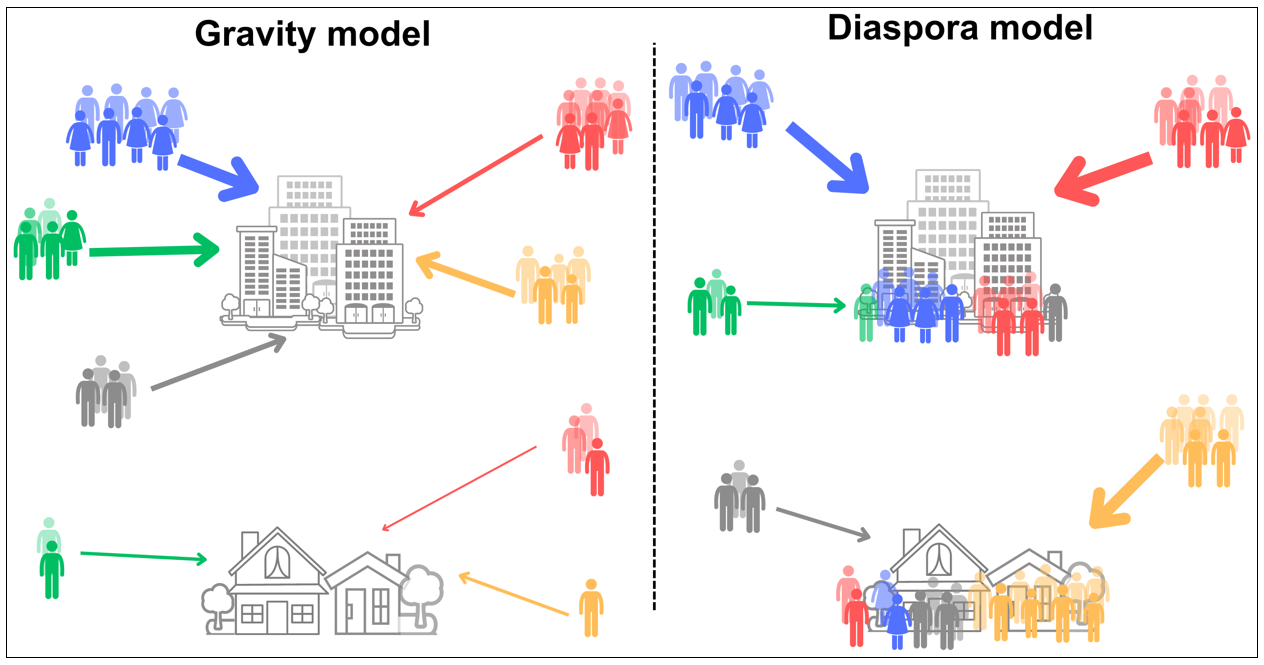

Supplement: pgae178_Supplementary_Data [file pgae178_supplementary_data.zip › PNASNEXUS-PNASNEXUS-2023-01187RR-s09.png]
